# Artificial Intelligence Models Reveal Sex-Specific Gene Expression in Aortic Valve Calcification

**DOI:** 10.1016/j.jacbts.2021.02.005

**Published:** 2021-04-14

**Authors:** Philip Sarajlic, Oscar Plunde, Anders Franco-Cereceda, Magnus Bäck

**Affiliations:** a Department of Medicine, Karolinska Institutet, Stockholm, Sweden; b Department of Molecular Medicine and Surgery, Karolinska Institutet, Stockholm, Sweden; c Theme Heart and Vessels, Division of Valvular and Coronary Disease, Karolinska University Hospital, Stockholm, Sweden

**Keywords:** artificial intelligence, aortic stenosis, calcification, sex differences, AI, artificial intelligence, AS, aortic stenosis, CABG, coronary artery bypass graft, ML, machine learning, PCA, principal component analysis

## Abstract

Male and female aortic stenosis patients have distinct valvular phenotypes, increasing the complexities in the evaluation of valvular pathophysiology. In this study, we present cutting-edge artificial intelligence analyses of transcriptome-wide array data from stenotic aortic valves to highlight differences in gene expression patterns between the sexes, using both sex-differentiated transcripts and unbiased gene selections. This approach enabled the development of efficient models with high predictive ability and determining the most significant sex-dependent contributors to calcification. In addition, analyses of function-related gene groups revealed enriched fibrotic pathways among female patients. Ultimately, we demonstrate that artificial intelligence models can be used to accurately predict aortic valve calcification by carefully analyzing sex-specific gene transcripts.

## Highlights

• Differences in the clinical presentation and physiology of aortic stenosis in men and women complicate the management of the condition.
• By combining traditional inferential statistics, artificial intelligence predictive modeling, and genetic pathway analysis, one can gain further insight into sex-specific gene expression patterns, potentially driving the valvular phenotype differences between the sexes.
• Results from this study, implementing a mixed and comprehensive methodological approach, offer a foundation for further exploration of potential drug targets.

## Visual Abstract

**Figure:**
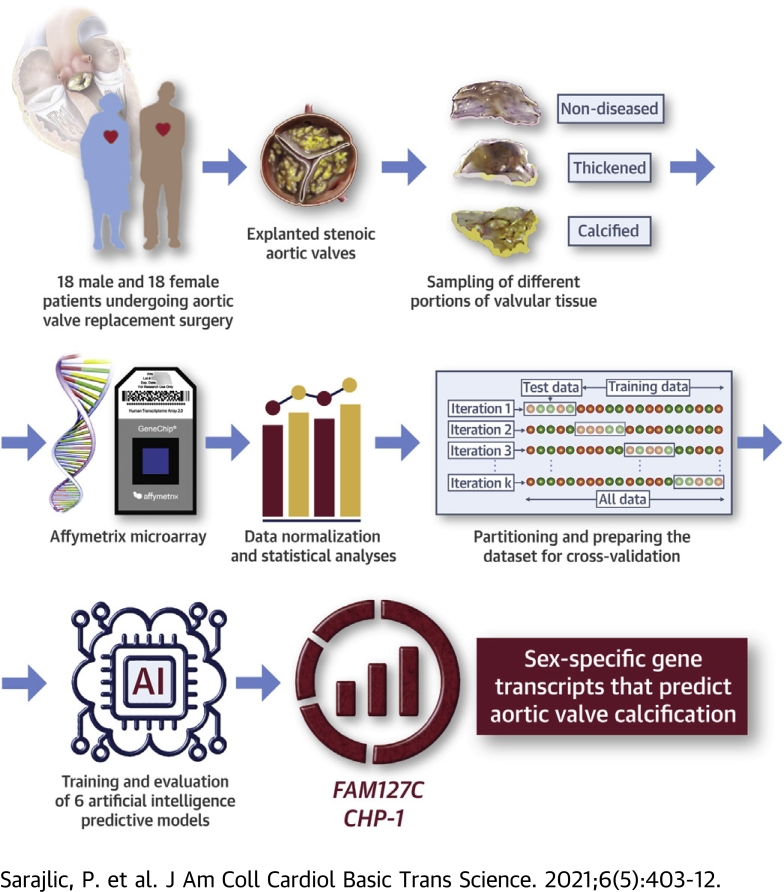

Aortic stenosis (AS) is the most common valvular heart disease requiring intervention in both men and women (1, 2, 3). However, male and female patients have a distinct phenotype of AS, which may lead to diagnostic difficulties in terms of evaluating aortic valve pathology in women (4, 5, 6). Likewise, the invasive either transcatheter aortic valve implantation or surgical aortic valve replacement (SAVR) rates remain substantially lower in women as compared with men (7).

The pathophysiology of AS is characterized by an inflammatory process due to endothelial damage caused by an interplay of lipid accumulation, mechanical stress, leaflet thickening, osteogenic differentiation, and ultimately calcification (8, 9, 10). A high-pressure gradient across the aortic valve arises as the aortic orifice becomes narrow and leaflet stiffness increases (11). When symptomatic AS is left untreated, the condition is rapidly fatal, leading to an adverse prognosis and high mortality (12). Recently, it has been shown that both moderate and severe AS carries a similar mortality rate (13). Although increasing attention has been paid to differences between the sexes in regard to cardiovascular diseases, there is a substantial lack of studies investigating sex differences in AS from a mechanistic point of view (14,15).

Gene expression studies have identified several target genes of interest when analyzing different pathophysiological processes in AS (16, 17, 18). However, none of these specifically addressed sex-specific transcriptomic patterns in AS. In addition, while traditional inferential statistics methods can offer great insights in datasets with a good observation (n) to predictor (p) ratio, there are multiple challenges in working with high dimensional datasets, such as gene expression data (19). Most regression models are known to underperform when there are ambiguities in how to select relevant predictors and when n < 10p, especially in situations where there are multiple nonlinear relationships (20). In addition, these datasets present with sparsity related challenges leading to an exponential increase in required data needed to detect significant findings. One solution to this problem is turning to artificial intelligence (AI) algorithms that are tuned for working with high-dimensional data and nonlinear relationships.

Regarding the management of AS, there are only a few distinguishments made between the sexes in the current guidelines despite emerging studies revealing possible physiological and clinical differences between male and female patients (21,22). For the same valvular calcification load, women tend to present with more severe AS symptoms (22). In addition, for the same anatomical degree of AS, women exhibit more concentric left ventricular geometry than men, and after surgical replacement of the aortic valve, there are sex-specific differences in left ventricular reverse remodeling (23). However, to our knowledge, there are no studies investigating sex differences in gene expression in AS. Therefore, the aim of the present study was to address this issue using valvular transcriptome-wide array data analyzed by cutting-edge methods to elucidate possible causes behind these clinical differences. The specific aims were: 1) to determine sex-specific differences in AS using novel statistical methods; and 2) to construct machine learning (ML) models to determine and quantify the ability to predict aortic valvular calcification with sex-specific gene expression data. To this end, we have implemented 2 subgroups of AI algorithms, ML, and deep learning.

## Methods

### Study population

Human aortic valves were obtained from patients undergoing surgical aortic valve replacement at Karolinska University Hospital in Stockholm. The cohort consisted of only patients with a tricuspid aortic valve. All patients gave informed consent, and the study was approved by the local ethics committee (2012/1633) and compliant with the declaration of Helsinki. Clinical characteristics of all patients were retrieved through electronic health records at the hospital and were recorded separately.

Study participants were matched by propensity scoring to minimize confounding effects based on clinical parameters across the groups of men and women (24). The following covariates were included in the matching model: age, concomitant coronary artery bypass grafting (CABG), body mass index, diabetes mellitus, smoking, and chronic kidney disease. Propensity score matching was performed before any analyses were conducted but after gene data were obtained from every sample. A total of 36 patients were included in the study, consisting of 18 men and 18 women.

### Valve preparation, RNA extraction, and gene expression analysis

Immediately after surgical removal, the valves were immersed in RNA Later (Qiagen, Hilden, Germany) and transported to the laboratory at 4°C. For each valve, 3 tissue samples were obtained based on the degree of aortic valve disease, and classified as nondiseased, intermediate, and calcified, according to previously defined criteria (25). In brief, macroscopically transparent and pliable valvular tissue was defined as nondiseased, whereas nonpliable, and nontransparent tissue was termed calcified. Thickened samples that were pliable but not transparent were termed intermediate.

RNeasy Lipid Tissue Mini kit (Qiagen) was used to extract RNA from valvular tissues using QIAzol Lysis Reagent. A microvolume spectrophotometer (NanoDrop, Thermo Fisher Scientific, Waltham, Massachusetts) was used to measure RNA concentrations and RNA quality was assessed with a 2100 Bioanalyzer (Agilent, Santa Clara, California). Only samples with an RNA integrity number >7 were kept for further analysis. cDNA was synthesized with the High Capacity RNA-to-cDNA Kit (Thermo Fisher Scientific). Genome-wide expression data were obtained for all 3 tissue types from each valve by using the Affymetrix Human Transcriptome Array 2.0. Affymetrix Transcriptome Analysis Console 4.0.2 (Thermo Fisher Scientific) was used to adjust between arrays ran in different batches, and data were normalized with signal space transformation-robust multichip analysis, yielding log2-transformed expression values.

### 2 × 2 plot

To assess basic differences between calcified and nondiseased valve tissue and between females and males, separate scores were calculated for tissue-type and sex by pairing data from 2 patients (1 from each group) after matching for the other parameter. Next, gene expression values for each gene were compared subject-wise and according to tissue-type. If gene expression was higher in the male compared to the female subject, a score of 1 was recorded for that particular gene and patient pair for the y-axis. Likewise, gene expression higher in the calcified part was recorded with a score of 1 for the x-axis. Means of the scores for the tissue-type and sex parameters of each gene in the dataset were shown on a 2 × 2 plot with the axes drawn at the center (at 0.5). Furthermore, analysis of enriched functional-related gene groups and pathways was conducted in the database for annotation, visualization, and integrated discovery.

### Sex chromosome gene expression

Expression of genes located on sex chromosomes may exhibit inherent differential expression between men and women. However, with the assumption that only those genes that add calcification predictability are relevant in terms of sex-specific aortic valve gene expression, expression levels of sex chromosome-located genes were explored as an unbiased gene selection towards a more guided pick of genes. Expression results for genes located on the sex chromosomes were extracted and used to predict if a given sample of the aortic valve was calcified or nondiseased.

### Statistical analysis

Data are presented as the mean ± SD, median (25th and 75th percentiles) or count (percentage). Standardized differences were used to determine if matching worked well between men and women. Data from transcriptomics experiments are traditionally assumed to follow either a negative binomial or Poisson distribution. These distributions can be approximated well by a normal distribution even in small sample sizes. Gene expression data were initially filtered by variance to remove genes that had little difference in expression levels across the groups of interest. The variance filtering was performed in conjunction with unsupervised ML, specifically, principal component analysis (PCA) and projection score optimization. To assess for differences in gene expression levels between calcified and nondiseased tissue samples and between the sexes, Student *t*-tests with false discovery rate correction (Benjamini–Hochberg) were used. Gene expression analyses between the tissues were regarded as repeated measures because the different samples came from the same patient. Two-tailed 95% confidence levels were used with q < 0.05 regarded as significant. In addition, filtering by fold change (FC) (between the degrees of calcification) was implemented where genes exhibiting 0.8 < FC < 1.2 were removed. To visualize raw gene expression results in a coherent way, unsupervised hierarchical clustering was implemented. The statistical software packages Qlucore 3.5 (Qlucore AB, Lund, Sweden) and Stata MP 16 (StataCorp LLC, 2019, Stata Statistical Software: Release 16. College Station, Texas) were used to conduct these analyses.

### AI models

RapidMiner Studio 9.6 (2019; RapidMiner, Inc., Boston, Massachusetts), and Python 3.8 (Python Software Foundation. Python Language Reference, version 3.8) was used to build the AI models. A nested approach was implemented, with 2 layers of abstraction, where a genetic algorithm was used as a heuristic search strategy that mimics the process of natural evolution to efficiently select the best predictors of calcification. The genetic algorithm contained a subprocess consisting of a random forest algorithm returning a performance vector after k-fold cross-validation. In k-fold cross-validation, the rows of the dataset are split into k partitions. For each partition, the model is first trained on it, and then, validated on the other partitions. This procedure is iterated over all partitions in the dataset and a measure of central tendency is reported for the average classification performance of tissue types across all folds. After careful consideration of our data, we chose a k-value of 6.

To compare the robustness of AI models and our data, 6 different ML/deep learning predictive models were created. The supervised learning models were: logistic regression, k-nearest neighbor, Naive Bayes, Gradient Boosted Trees, Random Forest, and Deep Learning networks. Default hyperparameter settings were used. The classification accuracy of each model was evaluated by comparing receiver operating characteristic curves.

## Results

The baseline characteristics of the cohort stratified by sex are summarized in Table 1. Lower standardized differences were achieved for the continuous variables that were included in the propensity matching algorithm than for the categorical variables. By matching for CABG an acceptable matching for prevalent cardiovascular disease was obtained across the sexes

**Table 1:** Clinical Characteristics of the Study Cohort Stratified by Sex

	Male (N = 18)			Female (N = 18)			Standardized Difference
	Mean ± SD	Median (Q1, Q3)		Mean ± SD	Median (Q1, Q3)		
Characteristics							
Age, yrs	75.3 ± 5.7	76 (72.9, 79.8)		74.4 ± 5.2	75.5 (72, 78.5)		0.16
BMI, kg/m^2^	29.1 ± 5.3	27.9 (25.6, 32.1)		29.4 ± 4.94	27.6 (26.0, 32.0)		0.08
MAP, mm Hg	97.8 ± 10.3	100.8 (90, 103.3)		94.5 ± 10	94 (86.6, 103.3)		0.33
HR, beats/min	71 ± 10∗	70 (62, 78)∗		75 ± 9	74 (70, 80)		0.36
CRP, mg/l	5 ± 6	2 (1, 6)		3 ± 3	1 (1, 3)		0.43
WBC, 10^9^/l	7.1 ± 1.9	7.2 (5.8, 7.9)		6.6 ± 1.9	6.5 (5, 7.4)		0.26
Hb, g/l	134 ± 11	133.5 (130, 143)		136 ± 15	139 (122, 150)∗		0.16
HbA1c, mmol/mol	42 ± 10∗	39 (34, 44)∗		38 ± 9	36 (35, 41)∗		0.34
eGFR, ml/min per1.73 m^2^	67 ± 22	73.5 (53, 83)		66 ± 15	61.5 (55, 82)		0.06
Categorical parameters							
Smoking							
Never			7 (39)			8 (47)	0.42
Former			11 (61)			8 (47)	
Current			0 (0)			1 (5.9)	
CABG			9 (50)			5 (28)	0.47
CVD			15 (83)			16 (89)	0.16
CAD			10 (56)			6 (33)	0.46
Diabetes			4 (22)			2 (11)	0.30
Statins			10 (56)			6 (33)	0.46

Values are mean ± SD, median (Q1, Q3), or n (%).

BMI = body mass index; CABG = coronary artery bypass graft; CAD = coronary artery disease; CRP = C-reactive protein; CVD = cardiovascular disease; eGFR = estimated glomerular filtration rate; Hb = hemoglobin; HbA1c = hemoglobin A1C; HR = heart rate; MAP = mean arterial pressure; WBC = white blood cells.

∗ N = 17.

**Figure 1:**
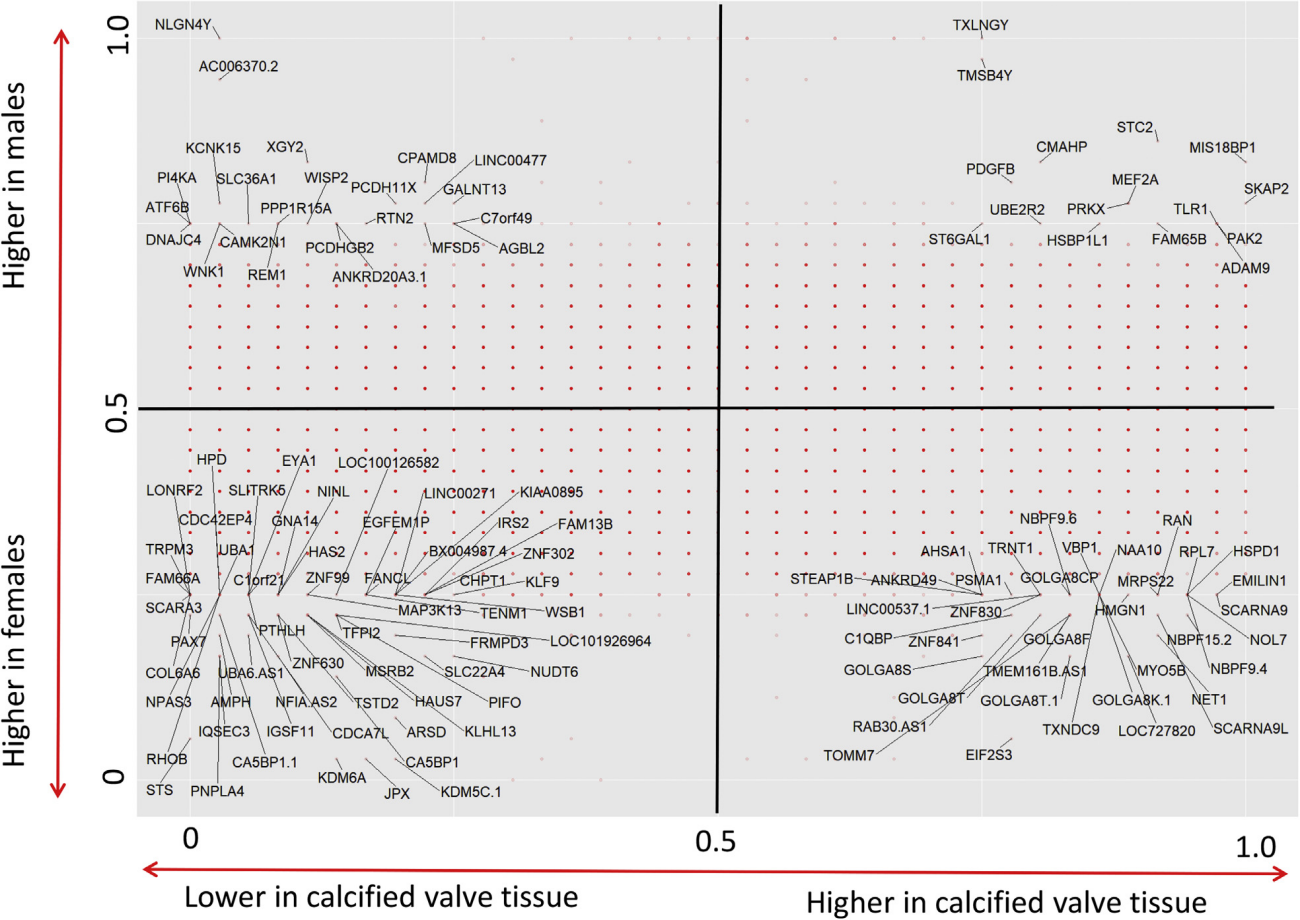
A 2 × 2 Plot A 2 × 2 plot indicating genes located at the 25% extremes of the tissue-type and sex axes. Dot-color opacity is correlated with the amount of genes located at a certain position in the chart.

**Figure 2:**
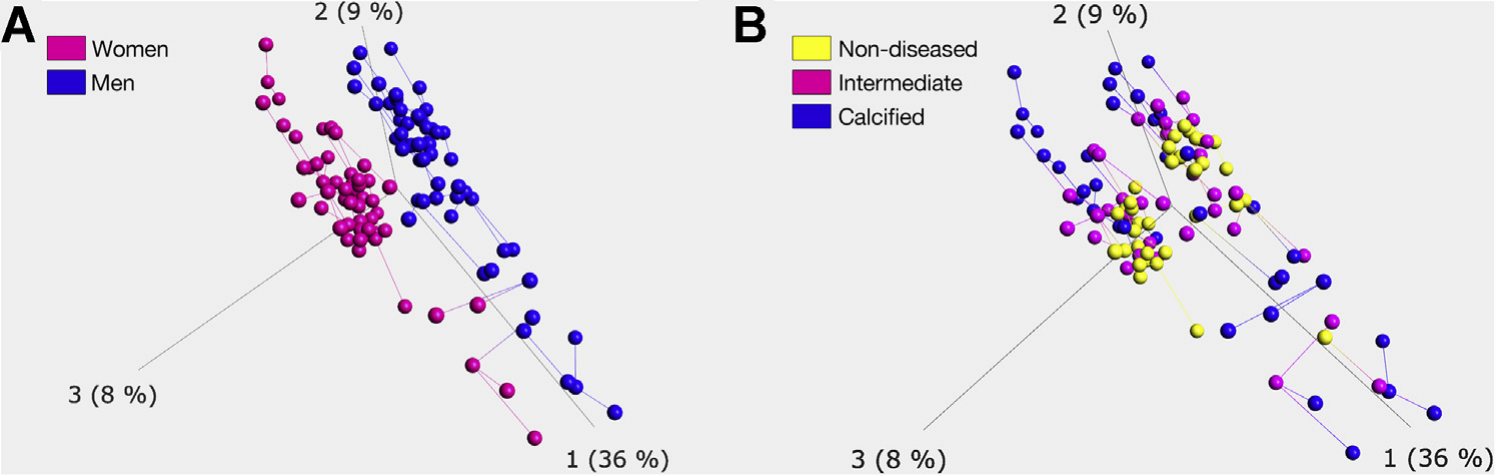
Principal Component Analysis Principal component analysis with filtering by variance and guided by projection score. **(A)** Colored according to sex (**pink** for women, **blue** for men). **(B)** Colored according to tissue type (**yellow** for nondiseased, **pink** for intermediate, and **blue** for calcified).

To get an overview of aortic valve gene expression patterns in males and females, means of scores for tissue-type and sex were shown on a 2 × 2 plot with the axes drawn at the at 0.5. Whereas most genes fell close to the axes (meaning similar distribution between the categories), a cut-off of the extreme 25% in each quadrant revealed a specific gene expression pattern separating calcified and nondiseased valve tissues in women and men (Figure 1). Pathways involving collagen protein metabolism and synthesis of elastic fibers (collagen type VI alpha 6 [COL6A6], elastin microfibril interface 1 [EMILIN1], scavenger receptor class A member 3 [SCARA3]) were found among the enriched gene sets in female patients (Figure 1). In further analysis for discovery of enriched functional-related gene groups and pathways through database for annotation, visualization, and integrated discovery, the collagen pathway formed a cluster with the highest enrichment score (1.36) among all gene sets analyzed in female subjects, whereas it was not enriched in men. PCA plots computed after filtering by variance achieved by optimizing the projection score showed cluster patterns for males and females (Figure 2A) across the different valvular calcification levels (Figure 2B).

After exclusion of genes with nonsignificantly different expression levels between calcified and nondiseased tissues, a total of 149 genes were identified to be differentially expressed between men and women after adjustment for tissue type (nondiseased, intermediate, calcified). The most differentially expressed genes between males and females in this analysis were X-inactive specific transcript (XIST) (log2(FC) = −11.8), tissue factor pathway inhibitor 2 (TFPI2) (log2(FC) = −0.81), taxilin gamma pseudogene, Y linked (TXLNGY) (log2(FC) = 4.21), and testis-specific transcript, Y linked 10 (TTTY10) (log2(FC) = 2.67). A heat map is shown in Supplemental Figure 1 and a volcano plot is shown in Supplemental Figure 2.

**Figure 3:**
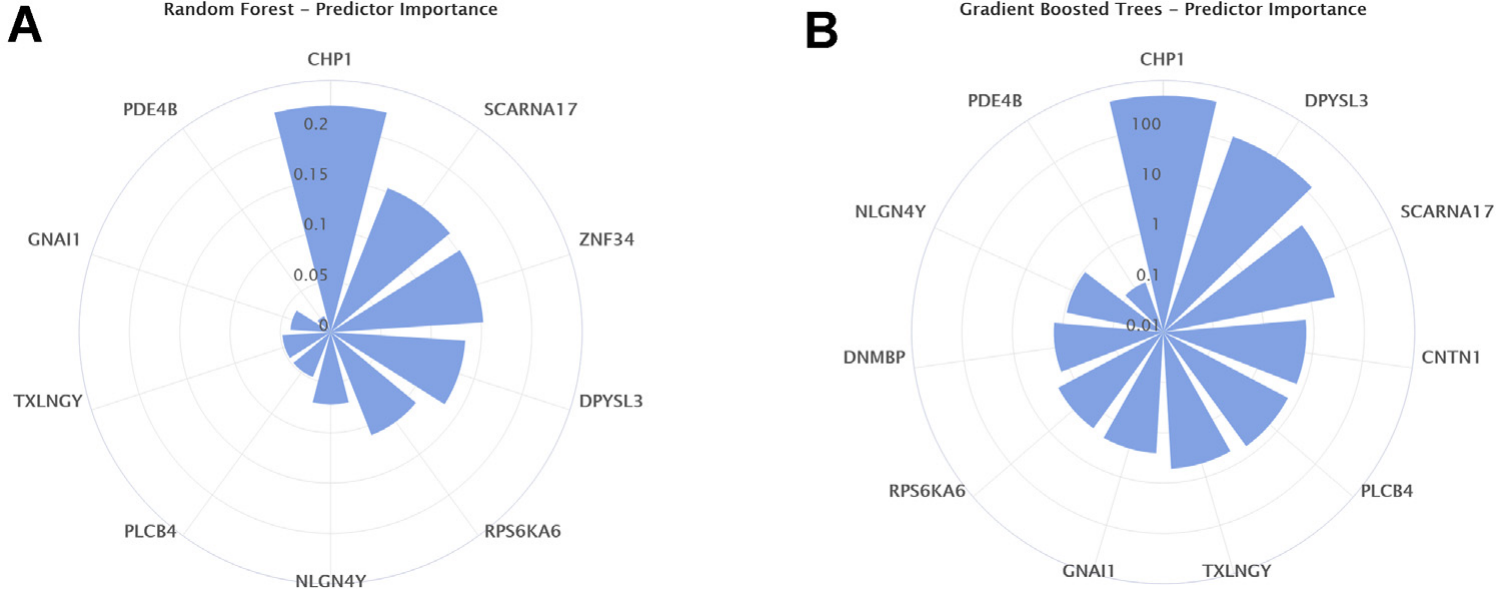
Genetic Predictor Importance Plots Polar plots showing predictor importance (weight) in the **(A)** random forest and **(B)** gradient boosted tree machine learning models using 149 differentially expressed genes between the sexes. A weight is given by the sum of improvements the selection of a given variable provided at a node.

Next, the 149 differentially expressed genes according to sex (Supplemental Figure 1) were used as input for either a logistic regression or ML models to assess calcification predictability. This process was performed to identify predictors independently of statistical significance and fold change. Logistic regression was significantly outperformed by all the other models, reaching a 100% accuracy rate after 6-fold cross-validation (Supplemental Figure 3). Predictor importance for the random forest and gradient boosted tree models are shown in Figure 3, where calcineurin like EF hand protein 1 (CHP1), small Cajal body specific RNA 17 (SCARNA17), zinc finger protein 34 (ZNF34), contactin 1 (CNTN1), and phospholipase C beta 4 (PLCB4) were some of the strongest predictors. These results were then compared with the chosen predictors by the naive Bayesian and k-nearest neighbor classifiers (Supplemental Table 1). To give a visual representation of identified gene expression cut-off values for some of the most important predictors, trees from the random forest model that had pure terminal nodes (i.e., trees that are not leading to any misclassification in the model) are shown in Supplemental Figure 4.

**Figure 4:**
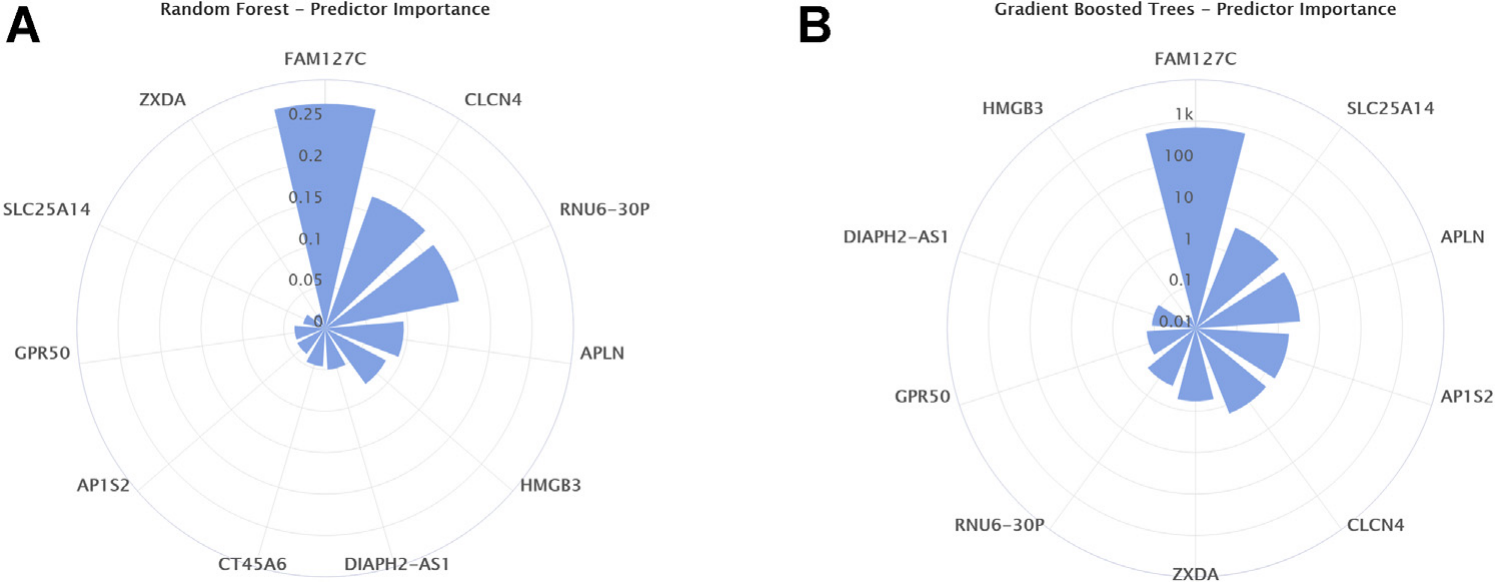
Sex Chromosome Gene Importance Plots Polar plots showing predictor importance (weight) in the random forest and gradient boosted tree machine learning models using all genes located on the sex chromosomes. A weight is given by the sum of improvements the selection of a given variable provided at a node.

Next, we extracted gene expression data from sex chromosomes only and implemented the same AI models as described above to assess the predictive ability of an unbiased gene selection. The results obtained were similar to the prior analysis with logistic regression being outperformed by all the other ML models reaching 100% accuracy (Supplemental Figure 5). These findings are summarized in Figure 4, and random forest models that had pure terminal nodes are shown in Supplemental Figure 6. Among the most important transcriptomic predictors of calcification were: family with sequence similarity 127, member C (FAM127C), apelin (APLN), and high mobility group, box 3 (HMBG3) (Figure 4) (Supplemental Table 2).

## Discussion

In this study, several different approaches were used to identify differential gene expression in male and female aortic stenosis. First, we show a particular sex-specific gene expression pattern across different degrees of calcification. Second, we selected 149 genes that were significantly different between calcified and nondiseased tissue samples and between males and females and applied machine learning to identify sex-differentiated transcripts that predicted calcification and which largely outperformed conventional multivariate analysis. Third, we show that valvular calcification can also be predicted by ML models, including valvular gene expression data from sex chromosomes only. Taken together, these results provide substantial evidence of sex-specific gene expression in the development of aortic valve calcification.

Whereas differences between male and female aortic stenosis are established in terms of calcification burden and clinical presentation (2,4,26), little is known about differences in valvular gene expression patterns. Separating individual valves into different degrees of structural changes, as previously described (27), allows to specifically study gene expression at different degrees of aortic valve disease (17,25). This is the first report showing qualitative differences in aortic valve gene expression between women and men that differentiate calcification. Comparing calcified and nondiseased valve tissue in men and women, the results support a more fibrotic calcific valve phenotype in women with AS based on collagen pathways being the highest enriched pathway. Considering all 3 valvular disease degrees, the present study subsequently identified a substantial amount of differentially expressed genes between the sexes. While the 2 × 2 and PCA plots provided a good overview of general trends in our dataset, it is important to acknowledge that this analysis does not consider the magnitudes, significance or validated predictability of gene expression differences across the groups of interest.

After the selection of transcripts whose levels were significantly different between calcified and nondiseased valve tissue, we identified 149 genes that were differentially expressed between males and females across the different categories of valvular disease. Subsequently, these differentially expressed genes between males and females were used to construct models for predicting valve calcification. Three major conclusions can be drawn from these analyses. First, whereas a logistic regression provided only poor prediction of calcification, all supervised ML models reached a prediction accuracy of 100%. Second, although the relative importance of the transcripts identified by the AI models differed somewhat between the models, there were some genes that were consistently included in the models. Third, the genes identified by AI were different from the ones identified by the largest FC. Taken together, this indicates that refining valvular gene expression analysis by AI may allow us to identify predictors of valve calcification.

Finally, expression levels of genes located on sex chromosomes were evaluated. Because these are genetically different between males and females, the predictability of their valvular expression levels for the valvular tissue phenotype further strengthen sex-dependent processes in valve calcification. Using the same methods as described above, all the AI models reached 100% predictability, indicating that sex chromosome valvular gene expression can also be used as source data for AI to predict valve calcification.

Of the genes identified in the different approaches, several encode proteins with potential biological importance for valve calcification. For example, one of the most down-regulated transcripts in males encodes tissue factor pathway inhibitor 2 (TFPI-2) (28, 29, 30), a serine proteinase inhibitor with anti-inflammatory characteristics regulated by calcification cascades in the aortic valve cells through interactions with matrix metalloproteinases and osteogenic markers (30). Likewise, X-inactive-specific transcript (XIST), also identified by FC difference between males and females, is involved in the regulation of sex-specific genes in both men and women and has been associated with calcification mechanisms in different tissues (31, 32, 33). Furthermore, many genes repeatedly identified by different AI algorithms to be preferentially expressed in female valves have potential mechanistic implications for valve calcification, including calcineurin (CHP1), phosphodiesterase (PDE4D), and matrix-remodeling (MXRA5) pathways. Several of the supervised ML models also indicated neuroligin-4 (NLGN4) as an important predictor of valvular calcification. The NLGN4 protein is implicated in autism spectrum disorders, but no studies have been conducted in cardiovascular tissues, showing the possible identification of novel sex-specific targets in valve calcification using the present approach. In the sex-chromosome focused analysis, all of the best performing models chose FAM127, a retrotransposon, as one of the most important predictive determinants of valvular calcification. Although there are only a few investigations that have been performed on this gene, 1 study provided causative evidence of retrotransposons inserting into the 3’ untranslated region of tumor necrosis factor, leading to the progression of valvular disease and chronic polyarthritis (34). Another important predictor identified in the present study was APLN, a vasoactive peptide that has been associated with AS (35).

A major strength of this study is that the amount of available data for analysis in the ML models was maximized by using a cross-validation strategy instead of a fixed split of the data into a training and validation set. This made it possible to ensure, in an efficient manner, that leakage of information was minimized. Taking multiple samples from the same valve specimen allowed for a thorough examination of different gene expression patterns across varying degrees of valvular calcification and fibrosis. In addition, combined analyses with both inferential statistics and supervised/unsupervised machine learning have, to our knowledge, not been done before in this setting.

### Study limitations

There are also some limitations of the study that should be acknowledged, the most notable being the relatively small sample size (n = 36). Alternative approaches to gene expression analysis, such as RNA sequencing, may offer a higher dynamic range than traditional microarrays. In the future, it would be beneficial to verify our results with such sequencing methods and to validate the findings by performing quantitative polymerase chain reaction in an independent cohort of patients. Despite matching of male and female patients, confounding from targeted variables cannot be completely ruled out. Finally, the observational design and lack of mechanistic explorations of the identified genes and pathways mean that no conclusion on causality can be drawn from the present study.

## Conclusions

In summary, the results from the multiple approaches in this study indicate that there are multiple combinations of sex-specific genes that can achieve good predictability of valvular calcification. By using different AI models, we were able to highlight the potential of these gene sets that might otherwise not be possible if only traditional statistics analyses and single models were used. The conclusions of the present study are that sex-specific valvular gene expression may underlie the differential phenotypes of AS in male and female patients, and that gene expression analyses beyond statistical significance and FC may expand the potentials produced by these datasets.

Perspectives **COMPETENCY IN MEDICAL KNOWLEDGE 1:** AS is one of the most common cardiovascular conditions with high mortality rates and no available medical treatment options.

**COMPETENCY IN MEDICAL KNOWLEDGE 2:** AS has particular sex-dependent features, in terms of risk, morphological changes in the stenotic valve, and disparities in treatment outcomes.

**TRANSLATIONAL OUTLOOK 1:** AI learning models can offer deeper insights into data where the ratio between variables and patients is high compared to traditional statistics approaches.

**TRANSLATIONAL OUTLOOK 2:** The results in this study identify several sex-specific candidate transcripts in valvular calcification that could offer a guide for future interventional testing of individualized medical therapies targeting critical pathways in aortic calcification.

## Funding Support and Author Disclosures

Supported by the Swedish Research Council (grant number 2019-01486), the Swedish Heart and Lung Foundation (grant number 20180571), the King Gustaf V and Queen Victoria Freemason Foundation, the Stockholm County Council (grant number 20170365), and the Marianne and Marcus Wallenberg Foundation (grant number 2015.0104). Dr. Sarajlic was supported by the Clinical Scientist Training Programme (CSTP) at Karolinska Institute. Dr. Franco-Cereceda was supported by a donation from Mr. Fredrik Lundberg. All other authors have reported that they have no relationships relevant to the contents of this paper to disclose.
