# Supplementary material for: Artificial Intelligence Models Reveal Sex-Specific Gene Expression in Aortic Valve Calcification
Source: JACC Basic Transl Sci. 2021 Apr 14;6(5):403–12. doi: 10.1016/j.jacbts.2021.02.005 (PMC8165113; doi:10.1016/j.jacbts.2021.02.005)

SUPPLEMENTARY MATERIAL

Supplementary Table 1. Top predictors of valvular calcification, chosen by the Naïve Bayes and k-Nearest Neighbor (k-NN) models, among 149 differentially expressed genes between the sexes.

Naïve Bayes	k-NN
TFPI2	CHP1
PDE4D	DPYSL3
NBPF11	TXLNGY
MXRA5	GNAI1
LONRF2	NLGN4Y
KLHL13	PDE4B
IL17RD	PLCB4
CHP1	RPS6KA6
CA5B	SCARNA17
	ZNF34

Supplementary Table 2. Top predictors of valvular calcification, chosen by the Naïve Bayes and k-Nearest Neighbor (k-NN) models, among all genes located on the sex chromosomes.

Naïve Bayes	k-NN
HS6ST2-AS1	HS6ST2-AS1
TMLHE-AS1	TMLHE-AS1
APLN	APLN
FAM127C	FAM127C
HMGB3	HMGB3
IL13RA1	IL13RA1
MIR891B	MIR891B
TRPC5	TRPC5

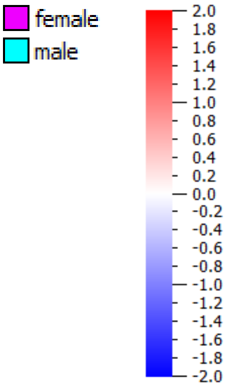

Supplementary Fig 1. Heatmap showing gene expression differences between the sexes. Genes with fold change values above 1.2 or below 0.8 (in regard to sex) that are significantly differentially expressed between the sexes and between non-diseased and calcified tissue samples are shown in the heatmap. The significance threshold was set at q<0.05 after Benjamini-Hochberg false discovery rate correction.

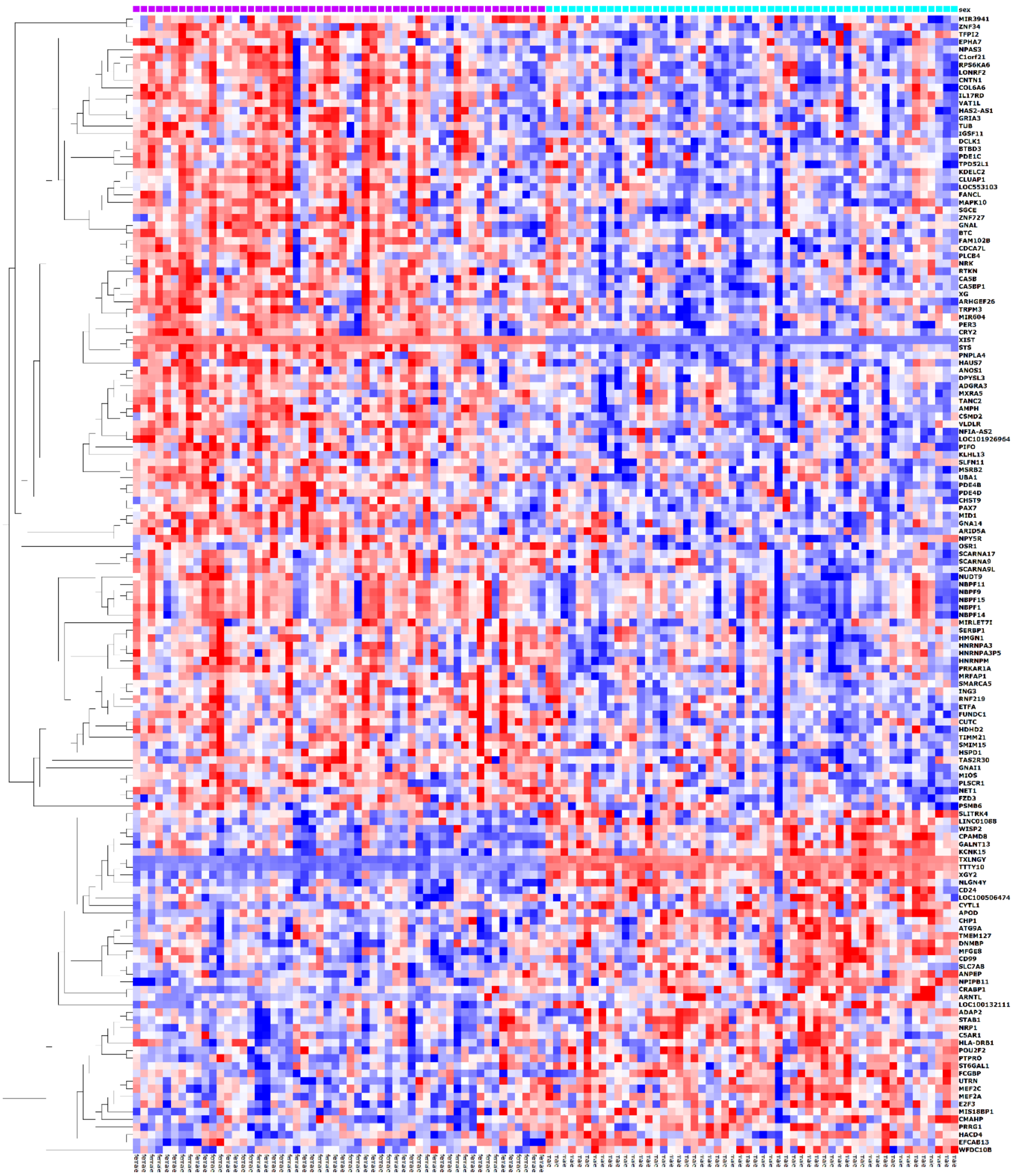

Supplementary Fig 2. Volcano plot showing the relationship between statistical significance and fold change in differentially expressed genes in men compared with women. TTTY10 (Log FC=2.67; -$\log_{10}$(p)=58.90) and TXLNGY (Log FC=4.21; -$\log_{10}$(p)= 84.21) are not shown.

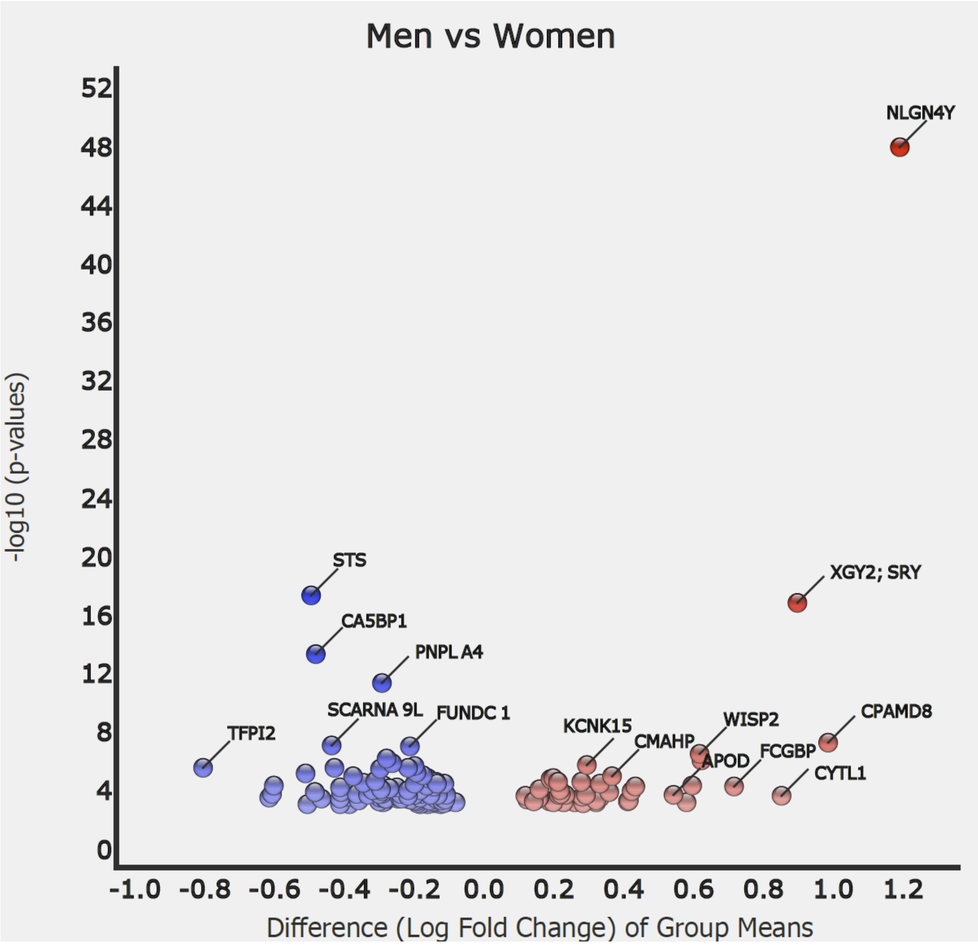

Supplementary Fig 3. Comparison of 6 machine learning models predicting tissue calcification based on 149 genes that were differentially expressed between the sexes.

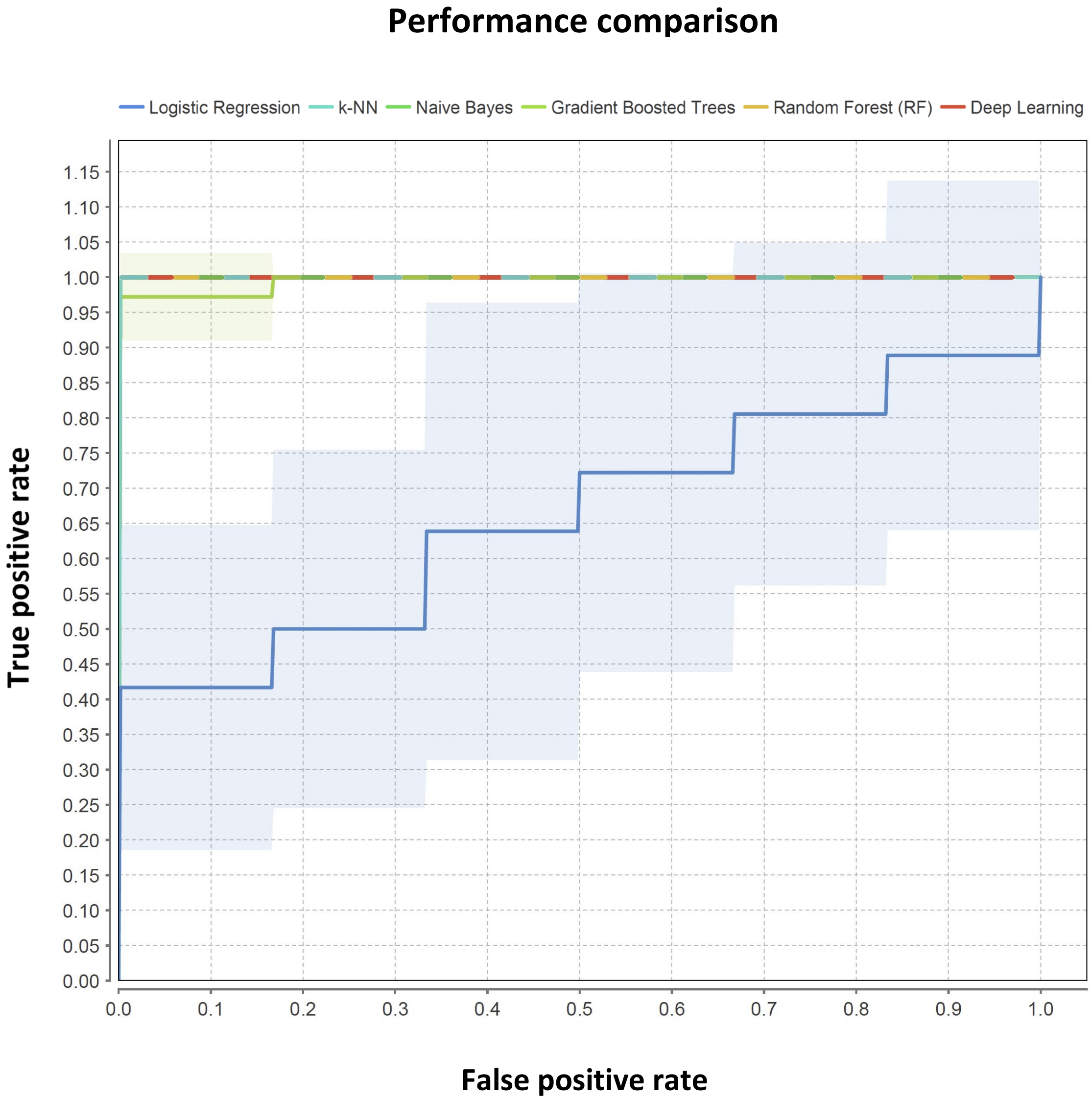

Supplementary Fig 4. Graphical representation of the random forest (RF) model predicting degrees of valvular calcification with trees containing pure terminal nodes, chosen based on predictor importance. The model was built by using the 149 identified genes differentially expressed between the sexes.

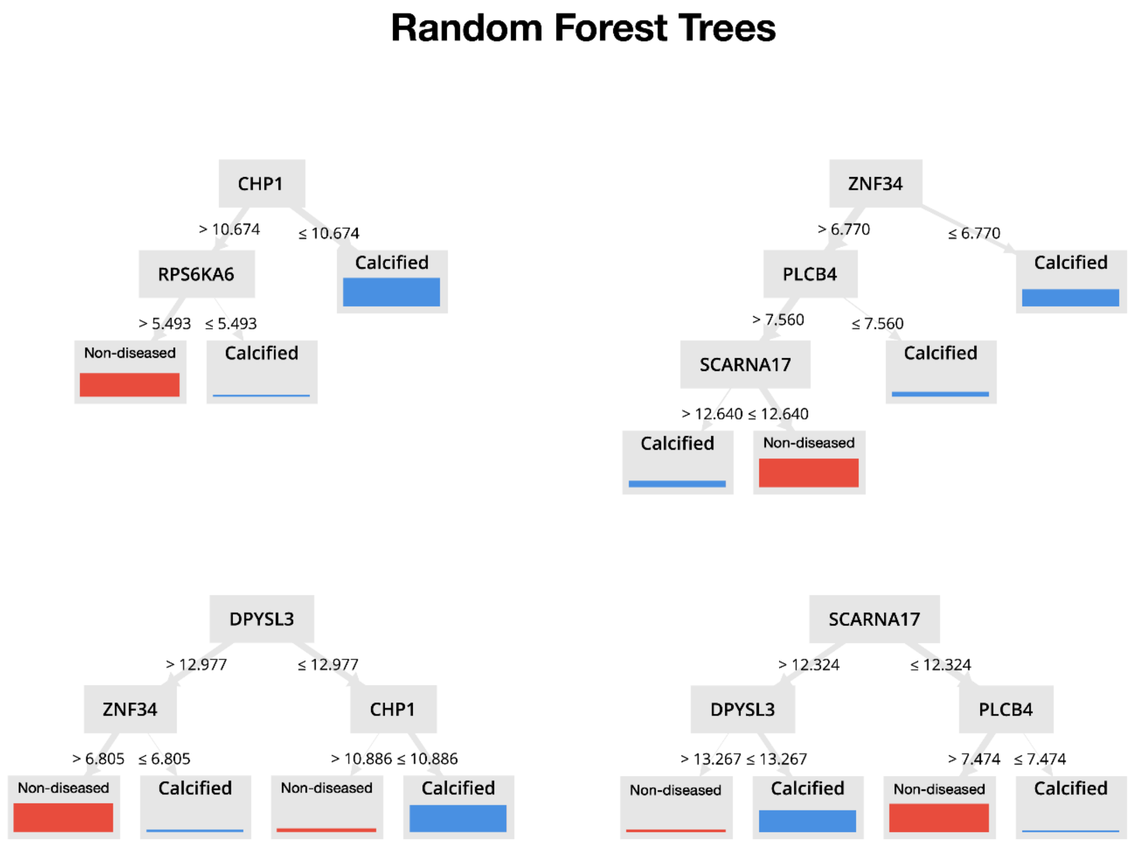

Supplementary Fig 5. Unbiased calcification prediction performance comparison between ML models by using all genes located on the sex chromosomes.

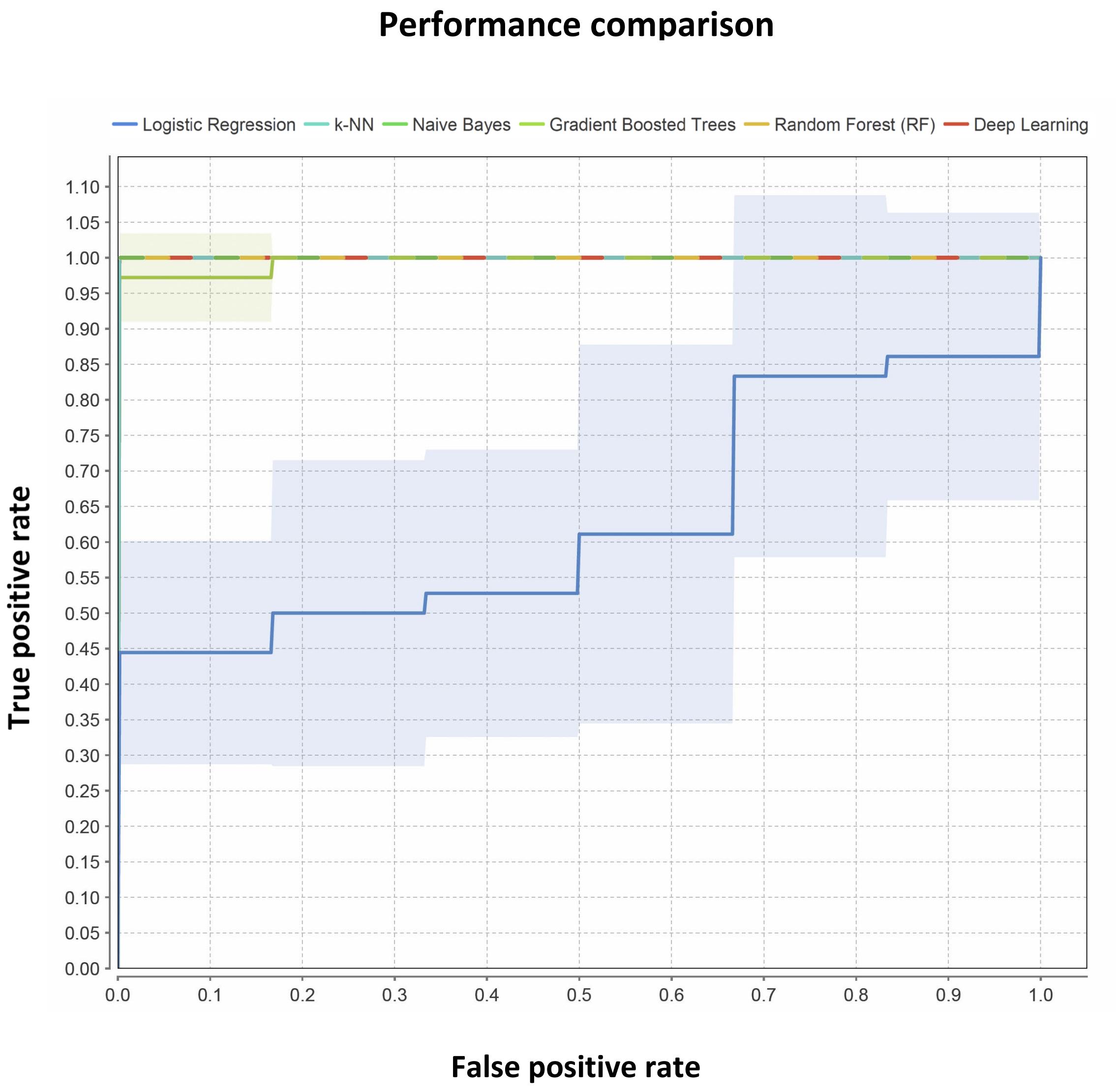

Supplementary Fig 6. Graphical representation of the random forest (RF) model predicting degrees of valvular calcification with trees containing pure terminal nodes, chosen based on predictor importance. The model was built by using all genes located on the sex chromosomes.

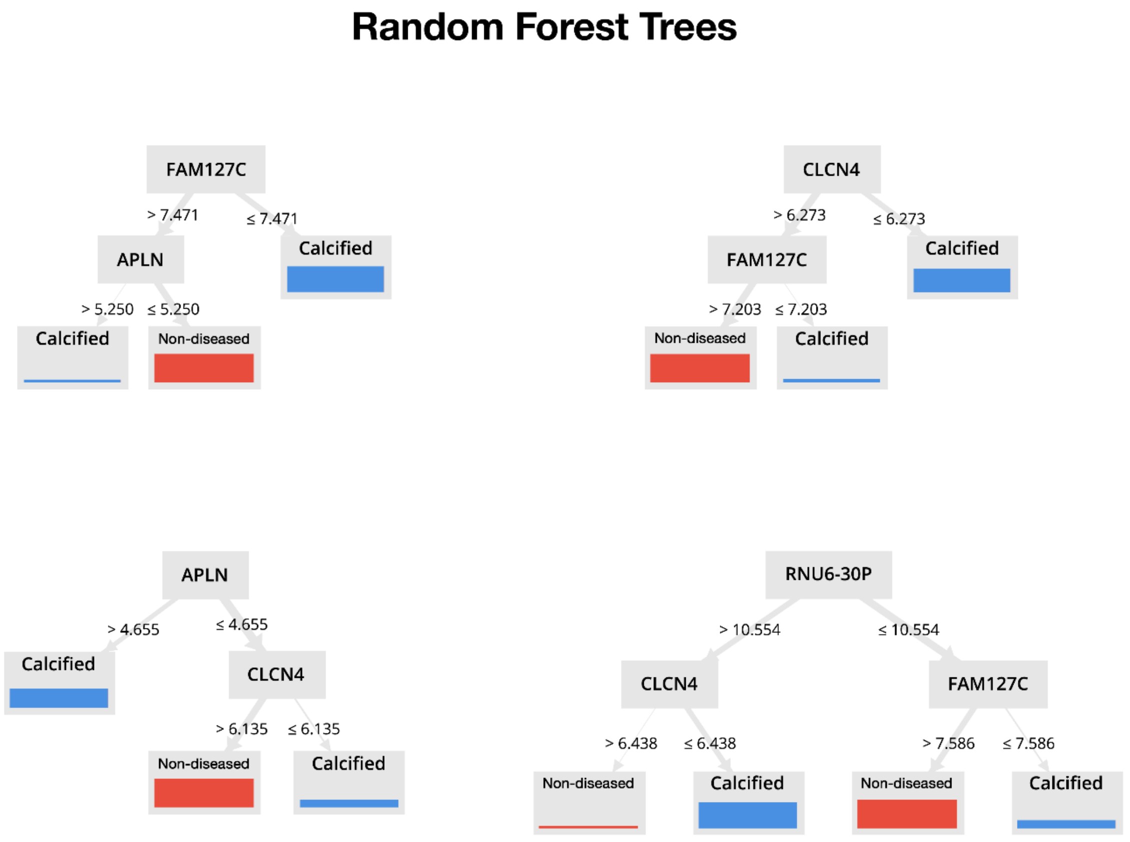

Supplement: Supplemental Figures 1–6 and Supplemental Tables 1 and 2 [file mmc1.pdf]
